# Anaesthetic efficacy and influence on cardiovascular parameters change of intraosseous computerised anaesthesia versus inferior alveolar nerve block anaesthesia in acute irreversible pulpitis of mandibular molars: study protocol for a prospective randomised controlled trial

**DOI:** 10.1186/s13063-022-06915-4

**Published:** 2022-12-05

**Authors:** Amany Laham, Roselyne Clouet, Gilles Amador del Valle, Alexis Gaudin, Tony Prud’homme

**Affiliations:** 1 Nantes Université, CHU Nantes, Unité d’Investigation Clinique en Odontologie, UIC 11, F-44000 Nantes, France; 2 Nantes Université, CHU Nantes, F-44000 Nantes, France; 3 Nantes Université, Oniris, CHU Nantes, INSERM, Regenerative Medicine and Skeleton, RMeS, UMR 1229, F-44000 Nantes, France

**Keywords:** Local anaesthesia, Dental anaesthesia, Analgesia, Irreversible pulpitis, Mandibular molars, Dental emergency, Pain management, Pulpotomy

## Abstract

**Background:**

Irreversible pulpitis is a highly painful inflammatory condition of the dental pulp. The recommended care is the removal of the coronal pulp (pulpotomy) for emergency treatment in multi-rooted teeth. However, achieving adequate analgesia in the case of mandibular molars presents many difficulties according to the dental literature. Intraosseous computerised anaesthesia (ICA), in particular, the QuickSleeper™ system, has already been demonstrated to be clinically efficient, but ICA is known to increase the heart rate reversibly in a manner that depends on the speed of injection. Therefore, accelerated heart rate was examined as a potential adverse effect of this injection. This trial aims to compare the cardiovascular effects and the anaesthetic efficacy of ICA versus inferior alveolar nerve block anaesthesia (IANB) in acute irreversible pulpitis of mandibular molars.

**Methods:**

This study is a non-inferiority prospective, randomised, controlled, single-blind, two-group trial conducted at the Nantes University Hospital. The study design included two parallel arms at a ratio of 1:1 that will allocate seventy-two patients into two groups: the first group will receive QuickSleeper™ intraosseous anaesthesia; the second will receive an inferior alveolar nerve block before emergency treatment (pulpotomy). This study will include patients requiring emergency dental care under local anaesthesia with confirmed irreversible pulpitis in mandibular molars. The primary outcome is the time taken for the fluctuations in the cardiovascular parameters to return to baseline values during endodontic treatment in two different anaesthetic techniques. The secondary outcomes include efficacy of the technique, patient’s pain perception before and after care, any post-operative effects, and factors associated with the delay of recovery of cardiovascular parameters between the 2 anaesthetic techniques.

**Discussion:**

This trial will evaluate the factors associated with heart rate increase during local anaesthesia administration. The use of blood pressure and cardiovascular measurements can be considered an asset and additional safety in managing local anaesthesia. Detecting early signs of potential adverse events (AEs), particularly patients with certain medical conditions (cardiac rhythm disorders), would suggest caution in administering anaesthesia.

**Trial registration:**

ClinicalTrials.gov ID: NCT03802305 registered January 14, 2019. (version no. 1.2; 5 November 2021)

**Supplementary Information:**

The online version contains supplementary material available at 10.1186/s13063-022-06915-4.

## Background

Irreversible pulpitis is based on subjective and objective findings that the inflamed vital pulp is unable to heal, as a result of bacterial infiltration [1]. The common symptoms include intense and paroxysmal spontaneous pain exacerbated by certain stimuli such as sweet, hot or cold foods and beverages [2]. However, relying on symptoms alone is not always clinically indicative of irreversible pulpitis because the tooth may occasionally appear asymptomatic and responds well to thermal testing. In such cases, dental history and thermal testing are the primary tools for assessing pulpal status. Furthermore, irreversible pulpitis is one of the most common reasons for emergency consultations in dentistry [2].

The treatment of choice is a pulpectomy, i.e. complete removal of pulp tissue. In an emergency appointment, however, due to time constraints, this treatment could be challenging to perform. In multirooted teeth, it is commonly accepted that a pulpotomy, within part of the pulp tissue, removed at the level of the dental crown, is performed. The purpose of an emergency pulpotomy is to stop the pain of pulpitis and results in a lower incidence of postoperative pain [3–6]. The standard anaesthetic technique is an inferior alveolar nerve block (IANB) by introducing the needle near the mandibular foramen at the spine of Spix.

Obtaining anaesthesia in patients with endodontic pain of pulpal origin is often difficult to achieve despite supplemental buccal infiltrations in addition to nerve blocks [7]. The failure rate is between 44 and 81% as was described in various clinical studies [8–10]. In addition, mandibular molars are the most difficult to anaesthetise [11]. In mandibular molars, there is an IANB failure rate of 15% up to 58% for non-inflammatory pulpitis [12, 13] and 44 to 81% for irreversible pulpitis [14–17]. IANB is performed blindly and guided only by anatomic landmarks that vary from individual to individual. Furthermore, there is a variation in the physicochemical characteristics such as lowered pH in the inflamed tissue that neutralises the base form of the anaesthetic in the nerve sheath and membrane. Furthermore, there are altered resting potentials and lowered thresholds of the excitability of the nerves in the inflamed tissue that leads to a varied state of innervation [18]. In addition, psychological factors (fear, anxiety) from one individual to another leads to a state of hyperalgesia or no analgesia that adds to the difficulties in achieving successful pulpal anaesthesia.

Anaesthetics called intra-osseous (IO) could be a more effective alternative. IO consists of depositing the anaesthetic solution into the alveolar bone supporting the teeth. In this method, anaesthesia begins immediately after the injection without causing soft tissue numbness. The injection scarcely causes any pain, and it requires relatively fewer amounts of the anaesthetic solution compared to conventional nerve block techniques [19, 20]. Several types of intraosseous anaesthetic system have been commercialised and studied clinically, such as the Wand® (Milestone Scientific, USA), the X-Tip (Dentsply, York, PA) and Stabident™ (Fairfax Dental Inc., Miami, FL) systems.

Several forms exist: intra-septal (IS), intra-diploid (ID), and more recently, intra-osseous computerised anaesthesia (ICA). However, IS administers the anaesthetic solution into the bone septum just after the cortex, a dense and reduced area, that may lead to partial or complete necrosis [21].

Intra-osseous computerised anaesthesia (ICA), such as the QuickSleeper™ system (Dental Hi-Tec, Cholet, France), administers the solution with a computerised control into the diploë (a richly vascularized bone) at a local rate and pressure. Thus, the QuickSleeper™ system is a form of ID in which the anaesthetic is delivered close to the root apices and associated periradicular tissues, leading to a greater reduction in periradicular sensation. The anaesthesia takes effect more rapidly since the solution is deposited close to the apex of the tooth at an axis of 15° to 30°. Thus, this form of delivery increases its effectiveness and reduces the risk of tissue necrosis, with better pain control (42 to 93% according to studies) [22, 23]. No soft tissue numbness occurs, and relatively fewer amounts of anaesthetic solution are needed. However, due to the fast diffusion of the anaesthetic solution in the diploe, the heart rate accelerates via the added effect of vasoconstrictors. This elevation is transient, and its effect is negligible and does not pose serious cardiovascular risk [12, 22].

There are currently many clinical studies that have evaluated cardiac parameters during IO administration. The heart rate increases in a reversible manner depending on the speed of injection [12]. The Stabident™ system showed a significant increase in heart rate of 8-28 beats/minute (BPM) on average with 2% lidocaine 1:100,000 epinephrine according to 3 authors [24–26]. By comparison, the X-Tip injection elevated the heart rate by 9–10 beats per min [27]. In regard to other parameters such as oxygen saturation and blood pressure, Pereira et al found no differences in either of them when using the X-Tip™ system but noted a significant increase in heart rate with 4% articaine (1:100,000 or 1:200,000 epinephrine) for anaesthetizing irreversible pulpitis lower molars [23]. Therefore, the accelerated heart rate could be a potential adverse effect of this injection [27].

Articaine is a common anaesthetic agent and a member of the amide family. The presence of its thiophene group increases its liposolubility, facilitating its diffusion into both nerve sheaths (such as the inferior alveolar nerve) and neural membranes of individual axons comprising a nerve trunk. The lower pKa of articaine would translate into a greater percentage of the drug in the active base form and its plasma protein binding is 95% higher than most other local anaesthetics [25–27].

The use of epinephrine allows an increase in the success of anaesthesia and a decrease in systemic toxicity, with better pain control. Rogers et al. published the first randomised, double-blind, clinical trial of the efficacy of 4% articaine versus 2% lidocaine in an IANB. They found that 4% articaine was significantly more effective than 2% lidocaine, with success rates of 62% and 37%, respectively [28].

There are even fewer studies on the QuickSleeper™ system and the variations in cardiovascular parameters (heart rate, systolic and diastolic blood pressure) that occur before and after anaesthesia administration. One study concluded that the increase in heart rate parameters caused by IO injection compared with IANB conventional block in impacted mandibular third molars was not significantly different [29]. However, there was no controlled concentration of epinephrine and no proper monitoring of the effects of anaesthesia.

### Objectives

The protocol mainly aims to compare the cardiovascular effects and the anaesthetic efficacy of intra-osseous computerised anaesthesia (ICA) versus inferior alveolar nerve block anaesthesia (IANB) in acute irreversible pulpitis of mandibular molars. The primary objective will be the evaluation of the time taken for the cardiovascular parameters to return to their initial values before administration of ICA compared to IANB in a mandibular molar presented with irreversible pulpitis. Secondary objectives will be the efficacy of the QuickSleeper™ system compared to IANB when (i) comparing patient pain, (ii) comparing postoperative outcomes, and (iii) investigating factors associated with the time to recovery of cardiovascular parameters between the 2 anaesthetic techniques. The use of blood pressure and cardiovascular measurements can be considered an asset and additional safety in the management of local anaesthesia through the early detection of signs of potential AEs. The main risk associated with local anaesthetics is a hypersensitivity or toxicity reaction, which is considered to be low leading to a very favourable benefit/risk balance.

## Methods/design

The trial protocol is identified as version n°2.0 of 11 December 2019. It adheres to Standard Protocol Items: Recommendations for Interventional Trials (SPIRIT) criteria; the SPIRIT Checklist can be found as Additional File 1: Table S1.

### Design

This noninferiority, prospective, randomised, controlled, single-blind, two-group study will be conducted in the emergency subunits of the Nantes University Hospital. Eligible patients with irreversible pulpitis in the first or second mandibular molars requiring emergency dental care will be included in the study. Diagnosis will be based on clinical and radiographic examination. Two parallel groups will be randomised at a ratio of 1:1 so that patients will either receive (1) intraosseous anaesthesia or (2) inferior alveolar nerve block anaesthesia. This manuscript used the SPIRIT reporting guidelines [30].

### Involvement in the design of the protocol

No members of the public or patient groups were involved in the design of the protocol.

### Participants

Adult patients seeking emergency consultation at the dental department of Nantes University Hospital will be included in the study according to the following criteria:

- Presence of spontaneous and/or diffuse pain with severe sensitivity to cold and/or heat tests in the first or second mandibular molar
- Patients over 18 years old (of any gender)
- Patients in good health, with no medical history (ASA 1 score) and no drug treatment for cardiac rhythm disorders (antiarrhythmics, beta-blockers)
- Patients able to give his/her oral consent.
- Affiliated with a health insurance
- Agree to be contacted by phone 72h after the emergency treatment

Participants will not be included if they present at least one of the following non-inclusion criteria:

- Contraindications to anaesthesia with vasoconstrictors
- Diagnosis of irreversible pulpitis of third mandibular molars, reversible pulpitis, acute apical periodontitis, periodontal lesion of endodontic origin or dentin syndrome
- Non retainable tooth requiring extraction
- Vital tooth serving as an abutment in fixed prosthesis
- Patients under the age of 18
- Adult patients who do not understand French or have refused to consent will not be able to participate
- Pregnant or breastfeeding women

### Outcomes

The main outcome of this study is to compare the time required in minutes for the cardiovascular parameters to return to their initial values between the two techniques: ICA with the QuickSleeper™ system versus the reference technique: inferior alveolar nerve block (IANB). The timing of the evolution of the parameters is based on 3 phases: “baseline” from T0 to T+4 minutes, “anaesthesia” from T+4 to T+8 minutes, and “pulpotomy” from T+8 to T+14 minutes. The return to baseline values will be defined as a success if 3 successive values of heart rate and blood pressure (systolic and diastolic) are identical to those recorded before injection. On the other hand, if there is an absence of 3 successive values of these two parameters, it is defined as a failure.

Secondary outcomes include:

- The O^2^ measurement performed during the 3 phases
- The efficacy of the anaesthesia with a VAS (visual analogue scale) of 0 and that the pulpotomy was completed successfully: the patient is asked about his or her perception of pain before the operation at D0 and D+1, D+2, and D+3
- Post-operative care
- Investigation of factors associated with delay in recovery of cardiovascular parameters (heart rate, dental anxiety, age, gender, and use of analgesics in the 6 h before surgery)

### Study timelines

**Fig. 1:**
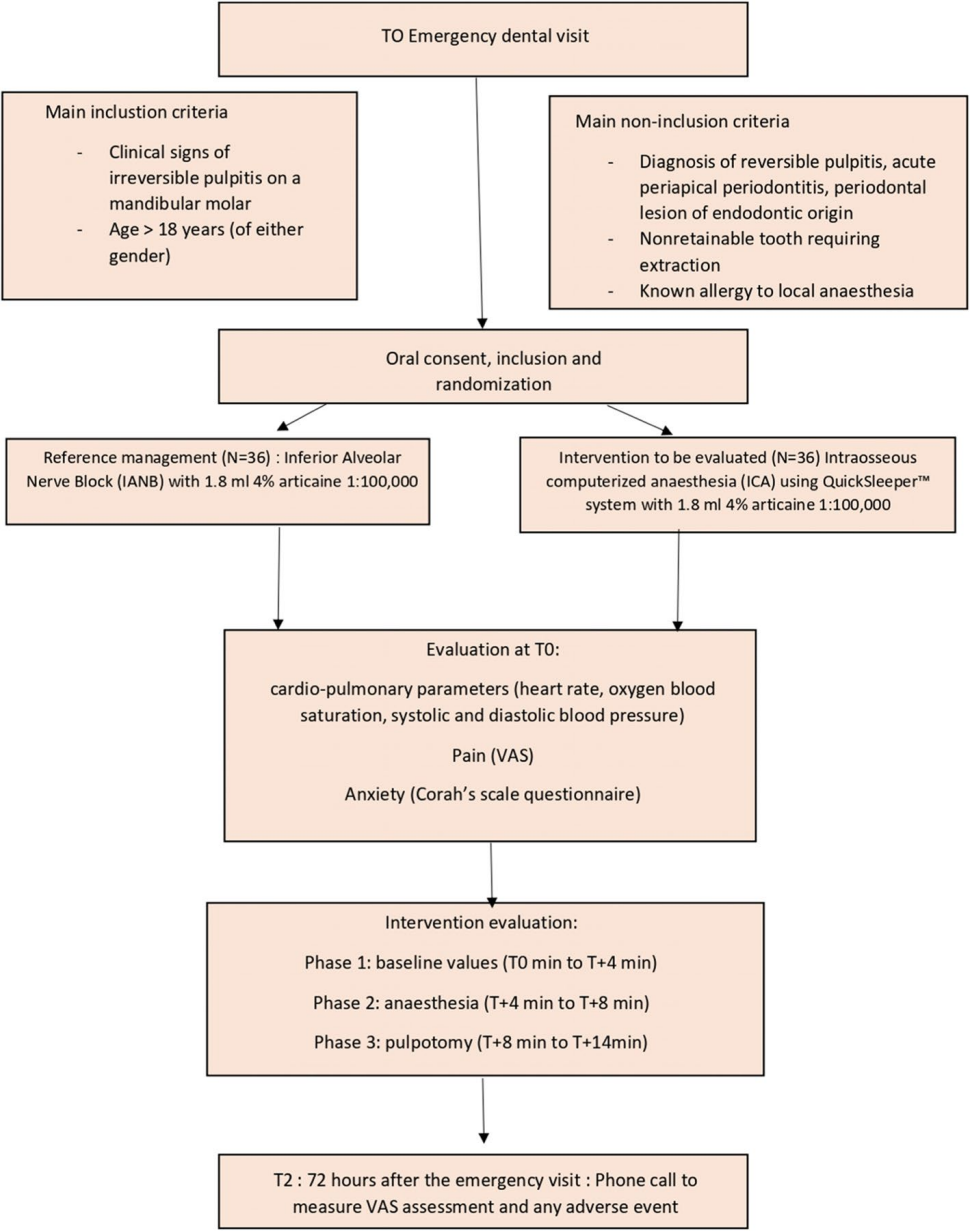
Flowchart of a trial evaluating the noninferiority of ICA QuickSleeper™ system versus IANB anaesthesia for the pain management in the emergency care of irreversible pulpitis in mandibular molars at the Nantes University Hospital

All patients seeking emergency consultation at the dental subunits of the Nantes University Hospital will be screened for the selection criteria. If a patient meets all criteria, he or she will be informed by an investigator and the investigation team of study technicians. He or she will be handed an information letter that comprises the objectives, methods, follow-up risks and restrictions of the trial. The patient’s oral consent is required before the start of any intervention and will be under the responsibility of the investigator to obtain consent. Thus, the patient’s consent will be obtained by the investigators only (Fig. 1).

The investigator in charge of the emergency room will proceed to randomisation (see below) using a secure computer program called Ennov Clinical and will conduct the emergency endodontic treatment. The patient will then be contacted by a research assistant 72h following the intervention by telephone to assess the pain perception and if any secondary effects of the anaesthesia occurred, as depicted in Table 1. Part or all of the study may be discontinued permanently or temporarily by the decision of the ANSM, the CPP, and the Study Sponsor (Nantes University Hospital).

**Table 1:**
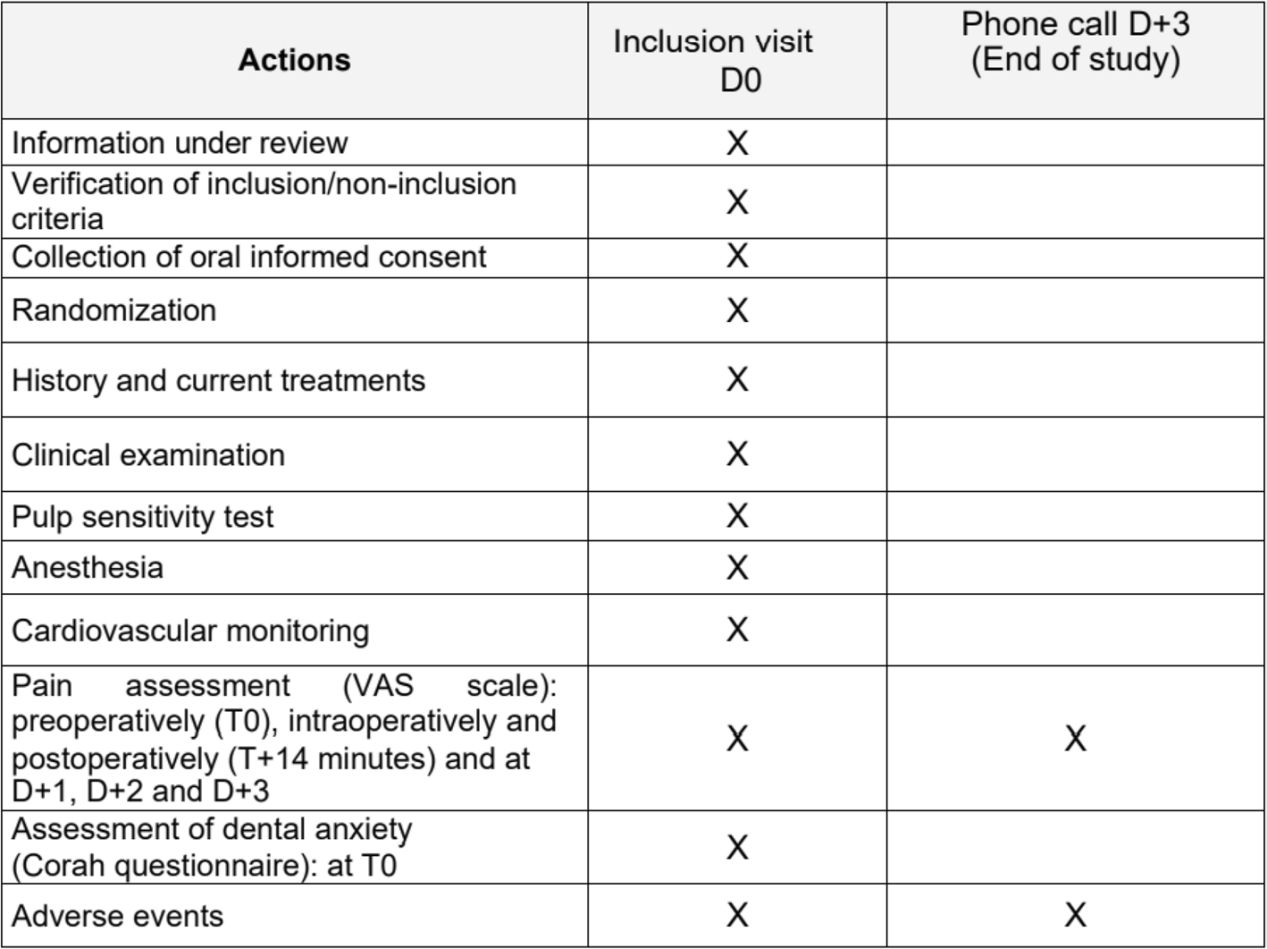
Schedule of enrolment, interventions, and assessments during COQ study protocol

### Auditing

The audit process will be done independently from investigators and the sponsor.

### Interventions

The patient will be blinded to the affected group and will fill out the first questionnaire which is the French version of Corah’s dental anxiety scale [31] to assess his or her anxiety about dental care. Then, the patient will be placed with a cuff on the arm, and a pulse oximeter on the other finger connected to a blood pressure monitor to monitor the cardiovascular parameters (heart rate, systolic and diastolic blood pressure, and oxygen saturation) throughout the intervention.

### Sequence generation

Each patient, regardless of the study group allocated, will receive 1.8 mL of 4% articaine 1:100 000 for 2 min. The evaluated intervention consists of the administration of intraosseous anaesthesia using the QuickSleeper™ (DHT, Cholet, France) system, according to the manufacturer’s recommendations, in the “ICA” group. A plastic syringe with a cartridge of 4% articaine 1:100,000 epinephrine will be used in the “IANB” group. The emergency treatment will be a pulpotomy. The tooth will be isolated with a rubber dam and all carious tissue and pulpal parenchyma will be removed. Pulpal bleeding will be controlled using a 2.5% sodium hypochlorite and the site will be covered with calcium hydroxide and a temporary filling. At the end of the intervention, all patients, regardless of the randomisation group, will be given two types of antalgics (paracetamol 1 g or paracetamol 600mg/codeine 50 mg) according to preoperative pain declaration (VAS) and recommended to take them only if they experience pain, according to the good clinical recommendations.

### Randomisation

Patients will be randomly assigned to one of the two arms: “ICA” and “IANB” at a ratio of 1:1. The randomisation list will be computer-generated by the study statistician and the randomisation process will be centralised through a secured clinical data processing website managed by Ennov© Clinical system software (Ennov, Paris, France). The investigators will enter the patient’s information (initials, age, and date of birth mentioning the month and year only), and the software will provide the patient’s unique allocation number and randomisation group.

### Allocation concealment mechanism

As it is a randomised controlled study, the patients are blinded. The unblinding occurs if a patient decides to leave the protocol prematurely. The best care will be provided by the investigator and the pulpotomy will be performed under the best conditions to relieve the patient’s pain.

### Determination of sample size

The sample size calculation was based on the noninferiority hypothesis in a pilot study that there will be an expected mean difference of 1 min 30 s between the two anaesthetic techniques with regard to the time taken for the values to return to the initial ones. According to the reported results from the few studies available, the proportion of patients having successful anaesthesia and better pain control in the reference group (intraosseous anaesthesia) would be 93%. The noninferiority margin was defined as 20%. The significance was set at 1.7% for the 3 main criteria (heart rate, systolic and diastolic blood pressure) and 5% for the secondary criteria. Thus, using a bilateral test with an epiR package 0.98-87 (Biostatgv), 72 patients (36 patients per arm) will be included in the study.

### Statistical analysis

The analysis will be based on intention-to-treat (ITT) using the “missing=failure” strategy. A robustness analysis, supported by the per-protocol approach, will be carried out if the patients present with protocol deviations (error of randomisation). Qualitative variables will be described by the percentages and numbers of each modality. Quantitative variables will be described by means and standard deviations. Confidence intervals at 95% will be presented.

A paper case report form (CRF) will be created for each patient inclusion for data collection and will be based on the establishment of a clinical database. All information required by the protocol must be provided in the CRF, necessary for statistical analyses.

To assess the primary endpoint (time to return to baseline heart rate and blood pressure), the mean values will be estimated by the Kaplan-Meier method (survival curves) and compared using Log-Rank tests. To assess the secondary endpoint (time to baseline O_2_ and change in measurements over time), the 2 arms will be compared in the same way as the heart rate and blood pressure parameters. Univariate and multivariate Cox models will be used to investigate the factors associated with the time to the baseline of cardiopulmonary constants. The difference in pain between D0 and D+3 will be compared between the 2 groups a Student’s *t*-test or a Wilcoxon test. Data for the analysis are collected from the first questionnaire (French version of Corah’s dental anxiety scale), the three phases of cardiopulmonary monitoring, the VAS assessment, and the postoperative questionnaire given to the patient at the end of the procedure. The information recorded on the postoperative questionnaire will be collected by a telephone call 3 days after the procedure. All the data collected will be typed and inserted into an Excel spreadsheet for data entry in a secure computer network of Nantes University Hospital. The investigative team, mainly the investigators as well as the study technicians involved in this study protocol will have access to the data collected.

In regard to the post-trial care, the patients will either continue their dental care with their appointed dentist, or in the case where they do not have one, they will be following appointments at the Nantes University Hospital.

A dissemination policy concerning the statistical data will be implemented to ensure that the official statistics are consistent, reliable, and comparable.

### Protocol violations

All protocol violations occurring after randomisation will be listed in the clinical report form (CRF) and tabulated by the subject. The final assignment of participants to the per-protocol analysis will be decided at a blinded protocol review meeting before locking the database.

### Adverse events

Considering the low risks and constraints of this study, no adverse event related to the trial is expected. In addition to the measurements of blood pressure and heart rate variations, which are the objectives of the present protocol, the suspected AEs related to the administration technique or anaesthesia will be traced in the CRF and analysed according to the study schedule by internal monitoring. They will not be notified to the sponsor, even if they are serious. Any adverse event is linked to a failure of anaesthesia.

## Discussion

Computerised intraosseous anaesthesia has gained considerable interest among practitioners in the last 25 years [32]. Wand® (Milestone Scientific, USA) was the first system used in 1997. What IO systems have in common is that the computer system controls the speed and pressure of the infiltration of the anaesthetic solution, to reduce pain, discomfort, and anxiety of the patient. Therefore, they are better alternatives to conventional bloc anaesthesia to counter IANB failure or as a first intention.

Vincent’s sign is the collateral effect of traditional IANB with ipsilateral lower lip anaesthesia, commonly used by clinicians as evidence of a successful anaesthetic procedure. However, this does not confirm that a successful pulpal anaesthesia has been obtained. Furthermore, accidental biting and burning of the lip and tongue (due to prolonged labial anaesthesia), as well as potential risks of mandibular nerve injury or a localised hematoma of the vascular lesion could occur. This further encumbers the advantages of this technique when comparing it to IO systems.

Very few valid clinical trials exist on the subject, most being realised with systems of intradiploid (ID) anaesthesia other than QuickSleeper™. Several studies have shown that supplemental Stabident™ ID injection, when added to IAN block and using local anaesthetics with vasoconstrictors, significantly increased the success of pulpal anaesthesia for 60 min [33].

One of the most important aspects of any primary anaesthetic technique is the pain experienced during the injection. This is particularly important for patients suffering from pain and stress due to an acute inflammation of the pulp. IANB, in particular, has been considered a fairly painful injection in the acute pulpitis situation. Owing to its asymmetric triple bevel, the needle tip in the QuickSleeper™ system makes possible a painless and easy perforation into the bone. The patient would report no pain or mild pain during perforation and solution deposition, despite the long wait for the agent to deposit. In addition, slow injection of more than a minute results in higher success rates [12, 22, 25]. The study by Özer has concluded that the pain levels after slow administration of QuickSleeper™ resulted in 82.5% success in mandibular third molars in assessing anaesthetic efficacy [29].

Furthermore, this technique has considerable expected benefits (patient comfort, rapidity of action, reproducibility). In addition, there is less risk of technical failures, such as a blocked or broken needle and the ability to retrieve the needle. The QuickSleeper™ system is also able to administer the anaesthetic solution at varying speeds, in the context of a para-apical or an intraligamentary injection with the same device regardless of the location, density, and resiliency of the soft tissues at the injection site.

Adverse effects of intraosseous anaesthesia are associated with an elevated heart rate (tachycardia, malaise). The main causes of an elevated heart rate are related to the patient's perception, fear, and anxiety, exacerbated by the symptoms of irreversible pulpitis or by the amount of vasoconstrictor used. Because this elevation is transient, it does not pose a serious cardiovascular risk, rendering its effect negligible. In patients whose medical condition suggests caution in administering epinephrine, mepivacaine may be an acceptable alternative [24]. The main objective of this trial will be to conclude that the time required for the cardiorespiratory parameters to return their initial values will be similar to the two anaesthetic techniques used. This study should provide better data about potential risk with ICA technique such as the QuickSleeper™ system in healthy or cardiovascular-risk patients.

This new approach in dentistry would increase the number of successful pulpal anaesthesia in emergency endodontic treatments by avoiding IANB failures and alleviating compliance of patients.

## Trial status

This trial was registered at ClinicalTrials.gov and this article is based on the 2nd version of the protocol published in December 2019. It was not submitted earlier to be published due to changes in the study status. The recruitments have begun beginning of March 2019 and due to the COVID-19 pandemic, the patient inclusions were delayed to resume by May 2020. The final inclusion will be terminated 1 July 2022.

## Protocol amendments

A second version of this protocol was conveyed in regard to the post-treatment visit (visit 2).

## Supplementary Information

**Additional file 1: Table S1.** A completed SPIRIT checklist (unless the structured study protocol template has been used), or checklist for another appropriate reporting guideline.

## Data Availability

Not applicable

## Abbreviations

ID: Intra-diploid
IS: Intra-septal
IO: Intraosseous
ICA: Intraosseous computerised anaesthesia
IANB: Inferior alveolar nerve block
BPM: Beats per minute
CPP: Comité de Protection des Personnes
VAS: Visual analogue scale
